# Cutaneous Manifestations in Pancreatic Diseases—A Review

**DOI:** 10.3390/jcm9082611

**Published:** 2020-08-12

**Authors:** Raluca Miulescu, Daniel Vasile Balaban, Florica Sandru, Mariana Jinga

**Affiliations:** 1 Dermatology Department, Elias University Emergency Hospital, 011461 Bucharest, Romania; miulescuraluca@yahoo.com (R.M.); florysandru@yahoo.com (F.S.); 2 Faculty of Medicine, “Carol Davila” University of Medicine and Pharmacy, 030167 Bucharest, Romania; mariana_jinga@hotmail.com; 3 Gastroenterology Department, “Dr. Carol Davila” Central Military Emergency University Hospital, 010825 Bucharest, Romania

**Keywords:** acute pancreatitis, chronic pancreatitis, pancreatic cancer, cutaneous, pancreatic panniculitis, acanthosis nigricans

## Abstract

Pancreatic pathology, comprising acute and chronic pancreatitis, autoimmune pancreatitis and pancreatic neoplasms, primarily presents with gastrointestinal symptoms and signs; however, it is well recognized that it can also associate a wide range of extra-digestive features. Among these systemic manifestations, cutaneous involvement plays an important role both as a diagnostic clue for the pancreatic disease itself and serving as a prognostic factor for the severity of the condition. Recognition of these cutaneous signs is, however, far from being satisfactory, all the more as some of them are relatively rare. In the current review, we discuss skin involvement in pancreatic diseases, referring to pancreatic panniculitis, cutaneous hemorrhagic manifestations, skin metastasis, acanthosis nigricans, livedo reticularis, necrolytic migratory erythema and cutaneous fistula. We highlight the clinical characteristics, treatment and prognostic value of these lesions. Better awareness among medical specialties other than dermatology is needed for detection of the skin clues associated with pancreatic pathology.

## 1. Introduction

Despite a high burden to health care systems and significantly impaired quality of life of patients [1,2], pancreatic pathology often comes second to other digestive diseases, with respect to addressing risk factors, early recognition and research activity [3].

Pancreatic diseases, represented by acute and chronic pancreatitis, autoimmune pancreatitis and the dreadful pancreatic cancer, most frequently present with digestive symptoms, but they can also be accompanied by cutaneous manifestations, sometimes even preceding the abdominal features [4]. Skin signs such as jaundice and pruritus, sometimes accompanied by scratching-induced lesions, may be frequently encountered in pancreatic illness that obstructs the bile flow. Moreover, particular skin signs can be seen in systemic diseases which include pancreatic involvement, such as hyperpigmentation in hemochromatosis, xanthoma or xanthelasma in patients with metabolic syndrome and fatty pancreas [5].

However, there are other, rare cutaneous signs that may occur in pancreatic pathology but which can signal the diagnosis or predict the severity of the disease. Several such cutaneous manifestations have been described in both inflammatory and neoplastic conditions of the pancreas—see Table 1 [6,7]. 

Despite being infrequent, awareness about these dermatological clues of pancreatic pathology is of utmost value when considering their diagnostic and prognostic value. Moreover, as many clinicians have not encountered these skin signs during their practice, they can easily miss the opportunity to detect them in a pancreatic patient. Regarding their prognostic role, it is interesting to note that, although several reports have described a poor outcome in the presence of cutaneous hemorrhagic signs [6], they are not incorporated into currently available risk stratification tools used for acute pancreatitis.

Several case-reports, case-series and reviews of the literature on specific cutaneous signs in pancreatic diseases have been published. Our aim was to update the current state of the literature in a narrative review on all cutaneous findings in pancreatic pathology in order to increase awareness on these skin clues as the presenting clinical feature or as severity and prognosis markers of the disease.

## 2. Cutaneous Manifestations in Pancreatic Diseases

### 2.1. Pancreatic Panniculitis

Pancreatic panniculitis (PP, pancreatic fat necrosis, enzymatic panniculitis) occurs in 2–3% of individuals with pancreatic disease [8]. It results from fat necrosis in the subcutaneous tissue usually of the extremities, and presents as erythematous nodules which may ulcerate and exudate. There are several case reports and literature reviews on PP describing its association with both benign and malignant pancreatic pathology [9].

It was first described by Chiari in 1883 [10], and, since then, it has been reported in both inflammatory conditions of the pancreas such as acute or chronic pancreatitis, and in pancreatic neoplasia. PP may present during the course of pancreatic disorder or may be diagnosed before the underlying disease [11].

PP has a male predominance and occurs more frequently in alcoholic patients [12]. 

The mechanism is not well understood. It is thought that release of pancreatic enzymes (trypsin, lipase, amylase) in the bloodstream leads to an increase in vessel permeability with consequent neutral fat hydrolysis to form glycerol and free fatty acids, and finally generating fat necrosis and inflammation. Pancreatic enzyme levels are increased in blood and skin lesions. As there have been reports of PP with normal serum enzyme levels [8,13], some have proposed that part of the patients may also have additional factors contributing to PP occurrence, such as enzyme deficiencies which makes individuals unable to degrade pancreatic enzymes; others have considered this a separate metabolic panniculitis, such as the one associated with alpha-1 antitrypsin deficiency [14]. Besides the enzymatic damage of the endothelium, immune complexes and release of inflammatory cytokines (particularly adipokines) have also been proposed as possible pathogenic mechanisms [15,16]. Not least, both skin and pancreatic inflammation share phospholipase A2 as mediator, which could be targeted as therapy in some inflammatory conditions [17]. 

Clinically, PP consists of disseminated, erythematous-violaceus, painful nodules, usually on upper or lower extremities, but which can also be located on trunk or scalp [18]. Sometimes, the nodules can ulcerate and remove a brown, oily, viscous material, as a result of fat necrosis. 

The differential diagnosis includes: Erythema nodosum, lupus panniculitis, sarcoidosis-related panniculitides and erythema induratum of Bazin/nodular vasculitis [19].

Laboratory workup may reveal serum pancreatic hyperenzymemia and imaging is used to detect the underlying pancreatic disease. If sampled, the skin lesions show the following histopathological features: Mixed septal/lobular panniculitis without vasculitis, necrosis of adipocytes with formation of “ghost adipocytes” (enucleated cells after coagulation necrosis) and calcium deposits [20]. 

Besides acute or chronic pancreatitis, PP can harbinger pancreatic neoplasia, both carcinoma (most frequently acinar cell type) and neuroendocrine tumors [21], and can precede the disease by several months [22]. Persistent or recurrent PP should always prompt screening of an occult pancreatic malignancy.

Some authors have also described the association between pancreatitis, panniculitis and polyarthritis, entitled PPP syndrome [23]. It is characterized by PP associated with intraosseous fat necrosis caused by the lypolitic enzymes. While the PPP triad usually includes pancreatitis as one of the diagnostic features, it has also been reported in the setting of pancreatic malignancy [24].

Treatment of PP consists of detecting and addressing the underlying pancreatic disease. Endoscopy or surgical therapy may be warranted, which usually leads to resolution of cutaneous lesions. Symptomatic relief with non-steroidal anti-inflammatory drugs is also recommended [25]. Some authors suggested that PP may also benefit from octreotide, a synthetic somatostatin-like polypeptide, which inhibits pancreatic enzyme production [26]. In the setting of pancreatic cancer, resolution on PP has been reported with chemotherapy [27].

### 2.2. Cutaneous Metastasis from Pancreatic Neoplasia—Umbilical (Sister Mary Joseph Nodule) and Non-Umbilical

Cutaneous metastasis from pancreatic cancer are a rare finding, the most frequent reported site being the umbilical region—the Sister Mary Joseph nodule. Other sites have also been reported—face, neck, scalp, temple, chin, axilla, chest, abdomen, buttocks or even scrotum [28]. 

Skin metastasis has been mostly reported in adenocarcinoma of the pancreas, but other histological subtypes have also been associated with cutaneous spread–adenosquamous cell carcinoma, mucinous cystadenocarcinoma, neuroendocrine carcinoma or VIP (vasoactive intestinal polypeptide) tumor [7,29,30].

Clinically, they usually present as a red-violaceous, indurated nodule or mass, or less often taking the form of a plaque, swelling or thickening of skin [7]. Differential diagnosis includes primary umbilical neoplasm, umbilical hernia or endometriosis, keloid or pyoderma gangrenosum [31]. 

A thorough clinical examination of the skin is warranted in patients with pancreatic cancer in order to recognize such cutaneous metastasis, as some have reported missed metastatic nodules on preoperative imaging, which would have altered patient management [32].

Skin metastasis can sometimes be the first clinical manifestation of the underlying malignancy [33,34,35,36,37] and is usually correlated with advanced, disseminated disease [7]. 

Treatment is based on multimodal oncological therapy including surgery, radiotherapy and chemotherapy. Prognosis is often poor, given the metastatic stage of the pancreatic malignancy. 

### 2.3. Acanthosis Nigricans

Acanthosis nigricans (AN) is a common skin condition characterized by hyperpigmented plaques, with papillomatous hyperkeratosis, giving a velvety texture, distributed symmetrically on intertriginous sites (neck, axillae) [38]. It can be associated with benign or malignant conditions [39].

AN is commonly a cutaneous manifestation of insulin resistance related disorders, such as diabetes mellitus or obesity [40]. In malignant AN, lesions are more severe and extensive, rapidly spreading and involve special sites (palms, soles); moreover, patients may have unexplained weight loss [40]. 

The pathogenesis of AN is hypothesized to be related to insulin-like growth factor (IGF) in patients with metabolic syndrome. There is also evidence on secretion of transforming growth factor alpha (TGF-alpha), epidermal growth factor (EGF) and fibroblast growth factor (FGF), which promote keratinocyte and fibroblast proliferation [41,42,43].

In patients with gastrointestinal adenocarcinomas (stomach most commonly but pancreas also), AN often manifests with pruritus, and sometimes even with cutaneous/mucosal papillomatosis [44,45]. Clinical examination can reveal other associated paraneoplastic dermatosis such as tripe palms or eruptive seborrheic keratoses—the Leser–Trélat sign [46]. Differential diagnosis of AN includes intertriginous granular parakeratosis, confluent and reticulated papillomatosis, Haber syndrome, Dowling–Degos disease or acropigmentation reticularis of Kitamura [47].

Treatment of AN consists of diagnosing and treating the underlying disease. In case of malignancy, lesions may regress after therapy of the tumor [48].

### 2.4. Necrolytic Migratory Erythema

Necrolytic migratory erythema (NME) is a characteristic cutaneous sign of glucagonoma. Glucagonoma is a functional neuroendocrine tumor of the pancreas, characterized by hypersecretion of glucagon, which leads to a combination of signs and symptoms known as glucagonoma syndrome. Glucagonoma syndrome represents the association between diabetes mellitus, stomatitis or glossitis or cheilitis, weight loss, anemia and NME [49].

NME was first reported in 1942 by Becker et al. in a diabetic patient with pancreatic tumor [50]. Since then, over 600 cases have been reported worldwide [49]. Most frequently, glucagonomas are sporadic, located in the pancreatic tail, but, in 20% of cases, can be associated with multiple endocrine neoplasia syndrome type 1 (MEN 1); glucagonomas are frequently metastatic at diagnosis [51]. Of note, NME has been reported in conditions other than glucagonoma, such as viral hepatitis B and myelodysplastic syndrome [52,53].

The pathogenesis of NME is still unclear. Some authors suggest that the glucagon itself induces the skin condition. On the other hand, amino acid or essential fatty acids deficiencies may lead to epidermal protein depletion and necrolysis. Other studies indicate low serum zinc levels. Not least, others have theorized the role of inflammatory mediators and liver dysfunction in NME [54,55]. 

Clinically, NME presents as enlarging and coalescing plaques, with central clearing, leaving residual lesions (with central induration and blisters, scales and crusts in periphery) [56]. The histopathological examination of skin biopsy samples is often inconclusive, revealing non-specific features [56]. Detection of the glucagonoma is based on imaging methods, with somatostatin receptor scintigraphy being the most accurate [49].

Treatment of NME consists of surgery or other ablative procedures of the tumor, with curative or debulking intent. Chemotherapy, somatostatin analogues and infusions of amino acids can also be beneficial [49,57].

### 2.5. Hemorrhagic Manifestations—Cullen’s Sign, Grey–Turner’s Sign and Fox’s Sign

Cullen’s sign and Grey–Turner’s sign are described in patients with acute pancreatitis. Clinically, these signs represent an ecchymotic discoloration, a superficial edema with bruising in the subcutaneous fatty tissue, located in the periumbilical region (Cullen’s sign) or along the flanks (Grey–Turner’s sign) [58]. Some authors suggested that these skin clues occur in 3% of patients with pancreatitis, and may predict the severity of pancreatitis and development of complications [6]. However, Cullen and Grey–Turner signs are not specific—there are also reports in extrapancreatic pathology such as liver disease, ruptured ectopic pregnancy, ruptured aortic aneurysm or hemorrhagic ascites [59,60,61,62]. Moreover, it can reveal a complication after interventional procedures, such as liver biopsy or ERCP (endoscopic retrograde cholangiopancreatography)-related perforation [63]. With regard to differential diagnosis, mimickers of Cullen’s sign have been reported in the literature, such as hematoma-like metastasis of melanoma [64].

Fox’s sign is another cutaneous sign described in acute pancreatitis, which is characterized by proximal thigh ecchymosis [65]. The mechanism consists in retroperitoneal leakage of the hemorrhagic ascites along the fascia of psoas and iliacus, under the inguinal ligament, up to subcutaneous tissue in the upper thigh [6].

Moreover associated with acute pancreatitis, there are reports of bluish ecchymosis of scrotum (Bryant’s sign) or around the axilla [59]. 

The mechanism behind the formation of these ecchymoses is represented by the subcutaneous infiltration with methemalbumin, a product of digested blood due to diffused pancreatic inflammation [59].

Recognizing these signs on clinical examination is important for diagnosing the underlying possible cause and, when related to pancreatic pathology, as an indicator for the severity of the pancreatitis flare.

### 2.6. Livedo Reticularis

Livedo reticularis (LR) is characterized by mottled cyanotic discoloration of the skin, with particular network pattern. This skin condition can be physiological (related to cold exposure), idiophatic or secondary. Its pathogenesis is related to alterations in blood flow, either by vascular changes, blood viscosity or embolism. As a consequence, pale areas alternate with cyanotic ones on the affected skin, usually in response to cold and resolving upon warming. 

Among the secondary forms, there is a variant of LR, also called Wazel’s sign, which has been reported in acute and chronic pancreatitis [66,67]. Secondary LR is usually patchy and asymmetrical. The mechanism in pancreatitis-related LR is thought to be related to trypsin-induced injury of the microvascular network [6]. 

Differential diagnosis includes livedoid vasculopathy, erythema ab igne, reticulated erythematous mucinosis or viral exanthems (erythema infectiosum) [68].

Pancreatitis-associated LR resolves with resolution of the underlying disease [69]. Unlike other cutaneous signs, LR has limited prognostic value and a low diagnostic yield for pancreatic pathology [70]. 

### 2.7. Cutaneous Fistula 

Cutaneous fistula can occur as a complication of acute pancreatitis-related fluid collections, or after pancreatic surgery. Postoperative fistulas are characterized by leakage of enzyme-rich pancreatic fluid, due to disruption of pancreatic duct. By definition, a clinically relevant postoperative pancreatic fistula requires the following criteria to be fulfilled: Drain output of any measurable volume of fluid, with an increased amylase level > 3 times the upper limit of normal and with a clinical impact/condition related directly to the fistula [71]. Clinically, fistulas may present with sepsis, hemodynamic and electrolyte disturbances or excoriations, particularly in those with high-output drainage. Management is usually conservative (topical skin protection, parenteral nutrition, octreotide, correction of nutritional/electrolyte deficiencies), but some cases may require surgery [72]. 

### 2.8. Other Cutaneous Signs Associated with Pancreatic Disease

Other skin signs reported in pancreas-related conditions or part of a syndrome with pancreatic involvement are summarized in Table 2 [73,74,75,76,77,78,79,80,81,82,83,84,85,86]. Some of them are presented as rare case reports or small case series; others such as Trousseau’s syndrome may be encountered relatively commonly in clinical practice. 

## 3. Discussion and Conclusions

While several skin signs have been described in pancreatic pathology, some of them having significant diagnostic or prognostic value, awareness among non-dermatologists needs improvement. As some of them are quite rare, clinicians may not even encounter a case during their practice. However, if working in a large volume pancreatology unit, the aforementioned skin signs might be common, if thoroughly looked for. On the other hand, there are some relatively frequent cutaneous signs associated with pancreatic disease, which makes them a potential diagnostic clue for general practitioners, gastroenterologists, intensive care specialists or surgeons. Moreover, regarding acute pancreatitis-associated skin signs, these can be immediately visible on o complete physical examination and can represent a simple, inexpensive indicator for the severity of the flare, unlike the currently available invasive and complicated risk stratification scores [5]. The importance of recognizing skin lesions associated with disorders of the pancreas is summarized Table 3. 

To our knowledge, our review is the first to assess a comprehensive list of skin manifestations, along with their diagnostic and prognostic value, in pancreatic pathology. A limitation of currently available research on skin lesions in patients with pancreatic pathology is lack of accurate prevalence data, as skin signs are often not recognized or not carefully checked in these patients. 

While rare, skin signs associated with pancreatic disease may lead to an early diagnosis or predict severity of the underlying illness. Clinicians should be aware of these cutaneous signs in pancreatic pathology in order to recognize them and appropriately direct management.

## Figures and Tables

**Table 1:** Cutaneous manifestations in pancreatic diseases.

Pancreatic panniculitis
Hemorrhagic manifestations—Cullen’s sign, Grey Turner’s sign, Fox’s sign
Cutaneous metastasis (umbilical—Sister Mary Joseph nodule and other sites)
Livedo reticularis (Walzel’s sign)
Acanthosis nigricans
Necrolytic migratory erythema
Cutaneous fistula
Other skin conditions associated with pancreatic pathology

**Table 2:** Other cutaneous signs associated with pancreatic pathology.

Skin Involvement/Condition	Clinical Characteristics	Pancreas-Related Disease
Hypertrichosis lanuginosa acquisita	Rapid growth of long, fine, unpigmented, lanugo-type hair, predominantly on the head and neck, in adulthood	Pancreatic islet cell carcinoma
Palmar fasciitis and polyarthritis syndrome (PFPAS)	Symmetric arthritis with flexion contractures and thickening of the palmar fascia	Pancreatic adenocarcinoma
Familial atypical mole and melanoma syndrome	Multiple, atypical, dysplastic nevi	Increased risk for pancreatic cancer
Trousseau’s syndrome	Migratory superficial thrombophlebitis	Pancreatic cancer
Peutz–Jeghers syndrome	Mucocutaneous pigmented macules	Increased risk for pancreatic cancer
Leser–Trélat sign	Rapid onset of multiple seborrheic keratoses	Pancreatic cancer
Attacks of flushing	Sensation of warmth and redness on the face and upper chest	Carcinoid syndrome
Dermatomyositis/ polymyositis	Distinctive skin rash associated with inflammatory myopathy	Pancreatic neoplasia
Pityriasis rotunda	Hyper-/hypopigmented, nummular well-demarcated, scaly plaques Trunk, extremities	Pancreatic cancer
Palmoplantar keratoderma	Excessive thickening of the epidermis of palms and soles	Pancreatic cancer
Papular mucinosis	Abnormal mucin deposition in the skin	Carcinoma of the pancreas
Aquagenic wrinkling	Skin wrinkling (usually palms and soles) upon immersion in water	Cystic fibrosis
Nutrient deficiency dermatitis of cystic fibrosis (CFNDD)	Periorificial erythematous annular papules that progress to desquamating plaques	Cystic fibrosis

PFPAS, palmar fasciitis and polyarthritis syndrome; CFNDD, nutrient deficiency dermatitis of cystic fibrosis.

**Table 3:** Importance of recognizing skin lesions in pancreatic diseases.

Sign	Diagnostic Clue	Prognostic Value
Necrolytic migratory erythema	+	−
Cullen, Grey Turner sign, Fox sign	+	+
Pancreatic panniculitis	+	−
Livedo reticularis	−	−
Cutaneous metastasis	+	+
Acanthosis nigricans	+	−
